# Hodgkin Lymphoma Associated with Common Variable Immunodeficiency: The Role of Early Diagnosis and Multidisciplinary Management

**DOI:** 10.3390/hematolrep17060065

**Published:** 2025-11-27

**Authors:** Dávid Tóthfalusi, Anita Gulyás, Anna Koncz, Éva Zöld, Árpád Illés, Zsófia Miltényi

**Affiliations:** 1 Division of Haematology, Department of Internal Medicine, Faculty of Medicine, University of Debrecen, 4032 Debrecen, Hungary; gulyas.anita@med.unideb.hu (A.G.); konczanna14@mailbox.unideb.hu (A.K.); illes.arpad@med.unideb.hu (Á.I.); miltenyi.zsofia@med.unideb.hu (Z.M.); 2 Doctoral School of Clinical Medicine, University of Debrecen, 4032 Debrecen, Hungary; 3 Department of Clinical Immunology, Institute of Internal Medicine, Faculty of Medicine, University of Debrecen, 4032 Debrecen, Hungary; zold.eva@med.unideb.hu

**Keywords:** common variable immunodeficiency, Hodgkin lymphoma, Epstein–Barr virus, immunodeficiency-associated lymphoma, targeted therapy, multidisciplinary management

## Abstract

**Background/Objectives**: Hodgkin lymphoma (HL) represents a rare but clinically significant complication in patients with Common Variable Immunodeficiency (CVID). Immune dysregulation, impaired viral control, and Epstein–Barr virus (EBV) infection may contribute to pathogenesis and adversely affect treatment tolerance. This case-based review aims to highlight the impact of early CVID recognition and multidisciplinary management on outcomes in CVID-associated HL. **Methods**: A retrospective screening of 224 patients with HL treated at our institution between 2010 and 2023 identified two individuals with CVID and EBV-positive HL. These cases are presented in detail and contextualized within a structured review of the published literature. **Results**: The first patient, diagnosed with CVID prior to HL onset, received immunoglobulin replacement and a modified chemotherapy regimen substituting bleomycin with brentuximab vedotin, resulting in sustained complete remission. The second patient, in whom CVID was recognized only after HL relapse, experienced recurrent infections, intolerance to therapy, and fatal disease progression despite treatment with brentuximab vedotin, checkpoint inhibition, and rituximab. The literature review revealed only eight comparable cases, underscoring the rarity and complexity of this association. **Conclusions**: Early identification of CVID facilitates infection control and enhances tolerance to HL therapy, thereby improving clinical outcomes. Multidisciplinary, individualized management and incorporation of targeted agents are pivotal in optimizing care for this vulnerable population.

## 1. Introduction

Common Variable Immunodeficiency (CVID) is the most prevalent symptomatic primary immunodeficiency. It is characterized by reduced levels of immunoglobulins (IgG, IgA, and/or IgM) with impaired antibody production. Although CVID is primarily considered a B-cell defect due to impaired differentiation into plasma cells, recent studies have identified additional cellular abnormalities [1,2]. Clinically, CVID presents with an increased susceptibility to infections, particularly of the respiratory tract, while a significant proportion of patients also develop autoimmune or inflammatory complications. The diagnosis requires exclusion of other causes of hypogammaglobulinemia, including combined immunodeficiencies and malignancies. A genetic cause is identified in only 10–20% of cases, although recent discoveries, such as CTLA4 haploinsufficiency, have led to reclassification of some previously diagnosed CVID patients [1,2].

CVID may be diagnosed at any age, though it is most frequently identified between 20 and 40 years, with approximately 20% of cases diagnosed before age 20 [3]. The etiology is complex, involving genetic, immunological, and possibly environmental factors. Recent studies highlight both genetic and epigenetic mechanisms contributing to pathogenesis [4].

The clinical spectrum is broad: while recurrent infections remain the hallmark, autoimmune complications such as cytopenias are common, and chronic lung disease—including granulomatous–lymphocytic interstitial lung disease (GLILD) and bronchiectasis—significantly impacts morbidity and mortality. Lymphoid hyperplasia, splenomegaly, and an increased risk of malignancies—especially non-Hodgkin lymphoma (NHL) and gastric carcinoma—further contribute to the disease burden [1,5].

Due to defective B-cell and T-cell function, CVID patients are especially susceptible to Epstein–Barr virus (EBV). EBV infects over 95% of the population and establishes lifelong latency in memory B cells. In CVID, defective immune surveillance predisposes to chronic or reactivated EBV infections, increasing the risk of lymphoproliferative disorders, including lymphoma [6,7].

Hodgkin lymphoma (HL) is a rare malignancy characterized by Reed–Sternberg (RS) cells in an inflammatory microenvironment. Despite a generally favorable prognosis in immunocompetent patients, HL has a bimodal age distribution and may be associated with immune dysregulation and Epstein–Barr virus (EBV) infection. A distinctive feature of classical HL is the amplification of the 9p24.1 chromosomal region, leading to overexpression of programmed cell death protein 1 (PD-1) ligands PD-L1 and PD-L2 on RS cells, which promotes immune evasion through the PD-1 pathway [8,9,10,11,12].

CVID patients are at increased risk of lymphoma, with NHL more common, while HL occurs less frequently (0.46–1.4%). In EBV-associated HL, viral genome is present in RS cells, where latent membrane proteins (LMP1, LMP2) mimic signaling pathways promoting survival and proliferation. Nevertheless, most CVID-associated lymphomas are of B-cell origin and EBV-negative, suggesting that mechanisms beyond EBV, such as chronic immune activation, also contribute [13,14,15].

Recent evidence further supports the concept that classical HL arising in immunodeficient hosts represents a biologically distinct entity. Alibrahim et al. recently provided a comprehensive review of immune-deficiency- and immune-dysregulation-associated EBV-positive classical Hodgkin lymphoma, integrating cases from primary immunodeficiencies, HIV infection, and post-transplant settings [16]. They identified common pathogenic themes, including EBV latency-II infection, impaired cytotoxic T-cell surveillance, and chronic immune activation. Importantly, they emphasized that patients with inborn errors of immunity, such as CVID or CTLA4- and LRBA-related disorders, share overlapping mechanisms predisposing people to EBV-driven lymphomagenesis. This framework underlines the clinical relevance of our present cases and the need for individualized, immunologically guided management in CVID-associated HL.

The primary aim of this case-based review is to analyze the clinical presentation, treatment response, and outcomes of HL in patients with CVID. While CVID is a recognized risk factor for lymphoproliferative disorders, the co-occurrence of HL and CVID is rare and not yet fully understood, with an estimated incidence of 0.46–1.4% [13,14]. Through the presentation of two complex cases of EBV-positive HL in CVID patients, it underscores the impact of early CVID diagnosis on chemotherapy tolerance, infection control, and achievement of disease remission.

In addition, it explores the potential role of intravenous immunoglobulin (IVIG) therapy in reducing infection rates and supporting chemotherapy outcomes in CVID-associated HL. The efficacy and safety of targeted therapies, such as brentuximab vedotin and immune checkpoint inhibitors, are also discussed in the context of immunodeficiency. A central theme of this case-based review is the importance of multidisciplinary, individualized management to optimize patient outcomes.

This rare association of EBV-positive HL and CVID is of substantial clinical relevance, representing one of the first detailed analyses of such cases. It addresses the unique challenges of managing HL in immunodeficient patients and highlights how early CVID recognition and IVIG therapy can improve treatment tolerance and remission rates. Taken together, these findings support the need to adapt existing HL treatment protocols—developed primarily for immunocompetent patients—to the specific risks of infection and immune dysfunction present in CVID.

## 2. Materials and Methods

A retrospective screening was performed of 224 patients diagnosed and treated for HL at the University of Debrecen between 1 January 2010, and 31 December 2023. The study was conducted in accordance with the Declaration of Helsinki and was approved by the Institutional Review Board of the University of Debrecen (DE RKEB/IKEB 6477-2023, 21 June 2023).

For all patients, clinical data, laboratory parameters, histopathological findings, and viral serology were reviewed, with specific attention to EBV and Cytomegalovirus (CMV) status. Given the established link between immune dysregulation and lymphoma, serum immunoglobulin levels (IgG, IgA, and IgM) were also evaluated to detect possible underlying immunodeficiencies. Immunoglobulin deficiency was defined as IgG < 7 g/L, IgA < 0.7 g/L, and IgM < 0.4 g/L, consistent with international diagnostic criteria for primary immunodeficiencies, including CVID.

Within this cohort, two patients were identified with profound hypogammaglobulinemia consistent with CVID who subsequently developed EBV-positive HL. These individuals are presented here as detailed case illustrations. Accordingly, the manuscript is structured as a narrative review with illustrative cases, supplemented by retrospective institutional data.

To place our findings in context, a structured literature review was conducted in PubMed, Scopus, and Web of Science using search terms related to CVID and HL. Eligible studies included English-language case reports, case series, and observational reports that provided individual-level clinical details of HL in CVID. Review articles were screened to identify additional primary cases. Reports not specifying HL, those lacking sufficient clinical information, and non-English publications were excluded. In total, eight published cases met these criteria and were included in the analysis alongside our two institutional cases.

## 3. Results

Among the 224 HL patients analyzed, two individuals were identified as having markedly reduced immunoglobulin levels consistent with CVID. The identification of CVID in these patients is of particular significance, as CVID is a known risk factor for lymphoproliferative disorders, including HL, albeit at a lower frequency compared to NHL. The presence of CVID in these two patients suggests that underlying immune dysfunction may have played a role in their HL development, potentially due to impaired B-cell function and defective immune surveillance against oncogenic viruses like EBV. Moreover, the presence of EBV positivity in both CVID-associated HL cases further reinforces the hypothesis that immune deficiency facilitates EBV-driven lymphomagenesis.

### 3.1. First Case

A 33-year-old male with type 1 diabetes mellitus had a long-standing history of recurrent respiratory tract infections and febrile illnesses. In 2022, persistently reduced serum immunoglobulin levels prompted further immunological evaluation. The patient exhibited markedly low IgG levels, repeatedly below the normal range for his age, accompanied by decreased IgA concentrations. He also demonstrated poor antibody responses to previous vaccinations, confirming impaired adaptive immunity. As he was older than four years at diagnosis, the age-related criteria for CVID were met. Other potential causes of hypogammaglobulinemia, including combined and secondary immunodeficiencies, were excluded. Immunophenotyping revealed a reduced number of switched memory B cells, further supporting the diagnosis. These findings were consistent with the International Consensus Document (ICON) guidelines for CVID, and regular IVIG replacement therapy was initiated [17].

Following initiation of IVIG therapy, the frequency of bacterial infections decreased; however, viral reactivations remained clinically significant. Serial polymerase chain reaction (PCR) monitoring demonstrated recurrent EBV and CMV viremia. While episodes were largely self-limiting, they coincided with periods of clinical deterioration and recurrent febrile illness, contributing to cumulative immune dysfunction. These findings highlighted the impaired viral control characteristic of CVID.

However, in January 2024, the patient developed fever, night sweats, weight loss, and progressive cervical and mediastinal lymphadenopathy. Laboratory evaluation revealed elevated inflammatory markers, and excisional lymph node biopsy confirmed HL, nodular sclerosis subtype. EBV-encoded RNA was detected in RS cells, confirming EBV positivity. Staging positron emission tomography–computed tomography (PET/CT) scan demonstrated widespread nodal and extranodal involvement, consistent with stage IV/B disease.

**Figure 1:**
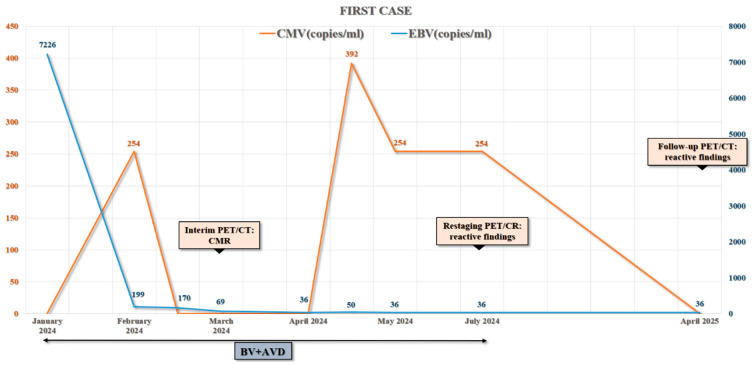
Longitudinal dynamics of Epstein–Barr virus (EBV) and Cytomegalovirus (CMV) viral loads in the first patient with common variable immunodeficiency (CVID)-associated Hodgkin lymphoma (HL). The graph depicts EBV and CMV viral copy number fluctuations over time in relation to the treatment period brentuximab vedotin (BV) + doxorubicin, vinblastine, and dacarbazine (AVD) and the results of interim, restaging, and follow-up PET/CT scans. Notably, EBV viral load shows a decline correlating with the initiation and course of HL therapy, whereas CMV reactivation occurred intermittently during treatment and follow-up.

**Figure 2:**
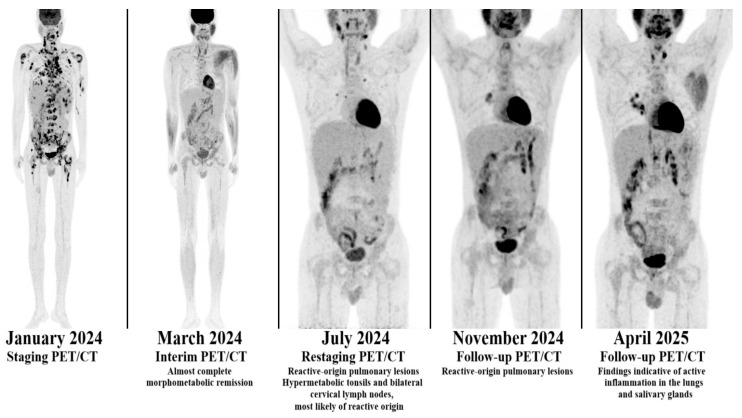
Positron emission tomography–computed tomography (PET/CT) images of the first patient with common variable immunodeficiency (CVID)-associated Hodgkin lymphoma (HL). Complete metabolic response (CMR) was achieved following treatment with brentuximab vedotin (BV) combined with doxorubicin, vinblastine and dacarbazine (AVD), reflecting effective disease control in response to tailored therapy.

Given the underlying immunodeficiency and the elevated risk of pulmonary toxicity, bleomycin was omitted from the standard doxorubicin, bleomycin, vinblastine, and dacarbazine (ABVD) regimen. Instead, the patient received brentuximab vedotin (BV) in combination with doxorubicin, vinblastine, and dacarbazine (AVD). Interim PET/CT after two cycles demonstrated complete metabolic remission (CMR), which was sustained at treatment completion. In the case of the first patient, a gradual decline in EBV viral load with intermittent CMV reactivations was documented during BV + AVD therapy, paralleled by PET/CT confirmation of CMR (Figure 1 and Figure 2). Importantly, IVIG therapy was continued throughout, ensuring improved infection control and enabling uninterrupted chemotherapy delivery.

As of September 2025, the patient remains in ongoing complete remission. This case highlights the dual role of EBV: not only as a pathogenetic driver of HL, but also as a recurrent systemic infection in the context of CVID, necessitating vigilant long-term monitoring and multidisciplinary management.

### 3.2. Second Case

Our 56-year-old male patient with hypertension-induced dilated cardiomyopathy and type 2 diabetes mellitus was first diagnosed with HL, nodular sclerosis subtype, in 2011. He was treated with six cycles of epirubicin, bleomycin, vinblastine, and dacarbazine (EBVD), which were well tolerated and resulted in complete remission as confirmed by post-treatment PET/CT. No intravenous immunoglobulin substitution was administered during this period, as hypogammaglobulinemia had not yet been recognized. The patient remained disease-free for nearly ten years.

In 2021, relapse occurred with progressive lymphadenopathy, B symptoms, and elevated inflammatory markers. PET/CT demonstrated widespread hypermetabolic lymphadenopathy. Second-line salvage chemotherapy (EBVD) was initiated, but interim PET/CT revealed only partial metabolic response with persistent disease activity. At this time, infectious complications began to increase, though no immunological evaluation was yet performed.

By early 2022, the patient showed clear disease progression with enlarging mediastinal and abdominal lymph nodes. He also developed recurrent febrile episodes and severe respiratory infections. Immunological assessment demonstrated markedly reduced IgG and IgA levels, consistent with a significant antibody deficiency. Retrospective review of earlier laboratory results revealed that hypogammaglobulinemia had already been present years before the onset of lymphoma but had not been further investigated at that time. Secondary causes of hypogammaglobulinemia were excluded, and the diagnosis of CVID was subsequently established. Regular IVIG replacement therapy was initiated but despite this, infectious complications persisted. Serial quantitative PCR monitoring revealed marked fluctuations in EBV-DNA copy numbers before initiation of therapy, consistent with intermittent, low-grade viral reactivation. This waxing-and-waning pattern closely parallels the kinetics observed in sporadic EBV-positive HL among immunocompetent individuals, in which transient peaks of viraemia reflect episodic reactivation from latently infected B cells under incomplete immune control rather than sustained viral replication. In our patient, the amplitude of these fluctuations was likely accentuated by the underlying hypogammaglobulinemia and impaired EBV-specific cytotoxic T-cell responses characteristic of CVID, resulting in prolonged viral persistence and enhanced inflammatory activity.

From mid-2022, several lines of salvage therapy were attempted. BV was administered, and interim PET/CT initially showed partial metabolic response. However, within months disease progression was again evident, and repeated EBV and CMV reactivations coincided with clinical deterioration. Repeat biopsy was performed during PET/CT-confirmed progression and histologically reconfirmed HL. In late 2022, therapy was switched to a PD-1 immune checkpoint inhibitor, resulting in temporary disease stabilization. Nevertheless, PET/CT after several cycles demonstrated persistent disease activity, and further EBV/CMV reactivations, together with bacterial pneumonias, undermined treatment efficacy.

**Figure 3:**
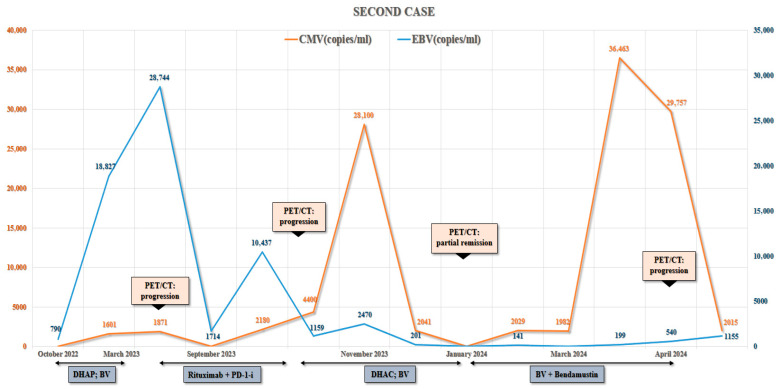
Longitudinal dynamics of Epstein–Barr virus (EBV) and cytomegalovirus (CMV) viral loads in the second patient with common variable immunodeficiency (CVID)-associated Hodgkin lymphoma (HL). The graph illustrates viral copy number fluctuations in relation to sequential treatment regimens, including dexamethasone, cytarabine, and cisplatin (DHAP) followed by brentuximab vedotin (BV), rituximab plus programmed death-1 inhibitor (Rituximab + PD-1-i), dexamethasone, cytarabine, and carboplatin (DHAC) followed by brentuximab vedotin (BV), and finally brentuximab vedotin combined with bendamustine (BV + Bendamustine). The outcomes of PET/CT scans (progression, partial remission) are also indicated. EBV and CMV viral loads demonstrate marked variability, correlating with disease progression, treatment response, and episodes of immune dysregulation.

**Figure 4:**
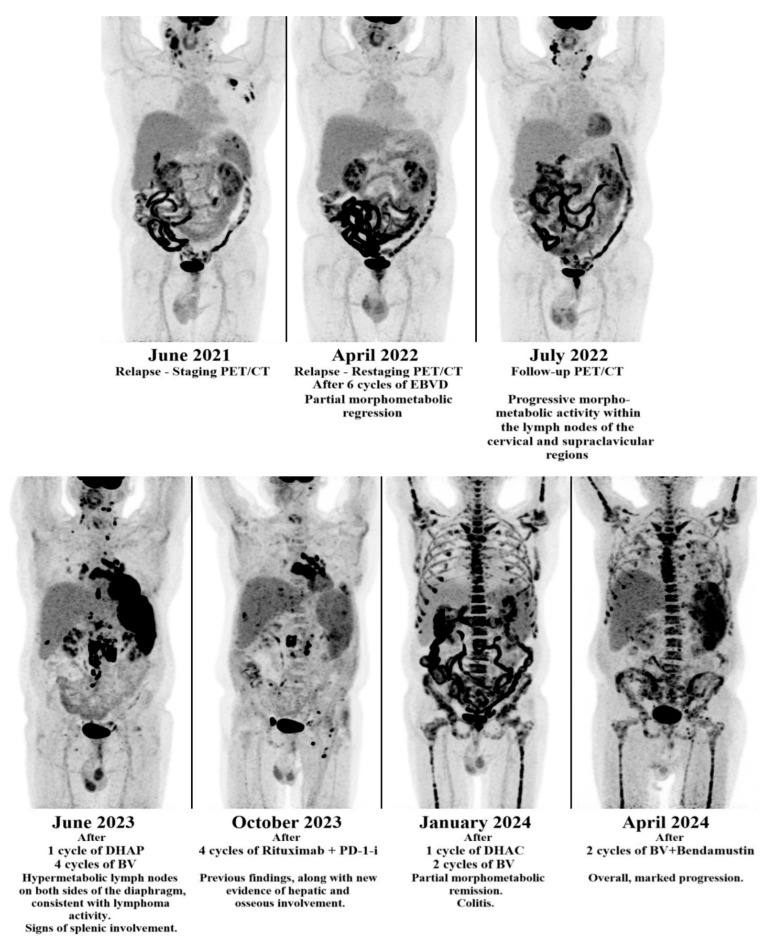
Positron emission tomography–computed tomography (PET/CT) scan results of the second patient with common variable immunodeficiency (CVID)-associated Hodgkin lymphoma (HL). The images demonstrate initial partial responses followed by disease progression despite multiple lines of therapy, including brentuximab vedotin (BV), programmed cell death protein 1 (PD-1) inhibitor, rituximab and chemotherapy. DHAP: dexamethasone, high-dose cytarabine and cisplatin; BV: brentuximab vedotin; PD-1-i: programmed cell death protein 1 inhibitor; DHAC: dexamethasone, cytarabine, carboplatin.

In early 2023, rituximab was introduced in an attempt to reduce EBV-driven B-cell proliferation. This intervention led to a transient decline in EBV viral load, but PET/CT confirmed continued lymphoma progression. Bendamustine was subsequently administered in mid-2023; however, the regimen was poorly tolerated due to severe cytopenias, recurrent infections, and worsening cardiac function, with no objective response observed. In the case of the second patient, EBV and CMV viral load fluctuations closely correlated with treatment responses, whereas PET/CT scans demonstrated an initial partial remission followed by progressive disease despite multiple lines of therapy (Figure 3 and Figure 4).

Over time, the patient’s condition worsened, and further oncologic therapy was no longer feasible. Supportive management focused on infection control, IVIG supplementation, and hematologic care. During hospitalization, he developed a generalized seizure, and neuroimaging revealed a lesion in the splenium of the corpus callosum. Differential diagnoses included lymphoma progression, hemophagocytic lymphohistiocytosis (HLH), and viral infection, but a definitive diagnosis could not be established due to thrombocytopenia. Comprehensive microbiological investigations, including serology and PCR assays for Toxoplasma gondii, JC virus, and Cryptococcus species, were negative.

Following the onset of neurological symptoms, his condition rapidly deteriorated, and he died on 1 May 2024. At autopsy, no histopathological evidence of opportunistic infection was identified in the central nervous system or other organs. Autopsy confirmed recurrent HL in the retroperitoneal lymph nodes, spleen, and liver, without central nervous system involvement. Extensive cardiovascular comorbidities, including dilated cardiomyopathy and severe atherosclerosis, were also present. The terminal heart failure was deemed secondary to underlying dilated cardiomyopathy and cumulative anthracycline-related cardiotoxicity, rather than to direct cardiac involvement by HL.

## 4. Discussion

Both of our cases illustrate the complexity of HL in patients with CVID, a rare but clinically significant association. In our institutional cohort, two EBV-positive HL cases were identified among 224 HL patients (0.89%), consistent with the estimated incidence of 0.46–1.4%. Although infrequent, HL in CVID is typically characterized by aggressive clinical courses and reduced treatment tolerance.

Due to the rarity and complexity of HL in the context of CVID, we conducted a comprehensive literature review to identify comparable cases. Our search yielded only eight reported cases [18,19,20,21,22,23,24], highlighting the limited available data and emphasizing the need for further investigation into this challenging condition.

### 4.1. Diagnosis and Timing

Our two cases underscore the decisive role of early CVID recognition. In the first case, diagnosis preceded HL, enabling IVIG initiation and more favorable infection control, which contributed to durable remission. In the second, CVID remained undiagnosed until HL relapse, by which point years of recurrent infections and comorbidities had already impaired tolerance and prognosis. Interestingly, his initial EBVD-based chemotherapy in 2011 had been well tolerated despite the absence of IVIG substitution, likely reflecting a subclinical phase of immune dysregulation that only manifested clinically years later as progressive hypogammaglobulinemia and recurrent infections. This contrast is reflected in the literature. Pediatric cases such as Özdemir and Tootoonchi involved early diagnosis, IVIG therapy, and ultimately favorable outcomes, comparable to our first patient [21,22]. Conversely, Ellwood reported an adult with CVID discovered only after HL manifestation, whose outcome mirrored our second case, with recurrent infections and poor treatment tolerance [19]. Likewise, Aghamohammadi et al. described two pediatric siblings in whom CVID was recognized late, after years of recurrent infections, and both had unfavorable outcomes, underscoring the prognostic impact of delayed diagnosis [24]. These comparisons highlight the importance of systematic immunoglobulin testing in HL patients with atypical presentations or recurrent infections.

### 4.2. Infection Control and the Role of EBV

Both of our patients were EBV-positive, consistent with prior findings that HL in immunodeficient hosts is frequently EBV-associated. In contrast, EBV status in published cases has been inconsistent: Rael et al. reported an EBV-positive case [20], Özdemir and Tootoonchi described EBV-negative pediatric patients [21,22], and the reports of Kishore, Ellwood, Aghamohammadi, and Tatci did not specify EBV status [18,19,23,24]. Therefore, the observed EBV positivity in our two cases should be interpreted as an association rather than proof of causality. Given the small number of available reports—including both EBV-positive and EBV-negative CVID–HL patients—it is plausible that multiple pathogenetic mechanisms contribute to lymphomagenesis in this setting. Impaired immune surveillance, chronic immune activation, and EBV reactivation may act synergistically, but the extent of EBV’s direct contribution remains uncertain. Early IVIG therapy in our first patient may have supported viral control and treatment tolerance, whereas delayed recognition of CVID and recurrent EBV/CMV reactivations in the second case coincided with poorer outcomes.

In the first patient, serial EBV-DNA measurements showed fluctuating viral loads before therapy, indicating recurrent episodes of low-grade reactivation followed by partial immune containment. Such waxing-and-waning kinetics are well documented in EBV-positive classical Hodgkin lymphoma of immunocompetent hosts and reflect transient viral reactivation from latently infected B cells rather than continuous viraemia. While this pattern is therefore not unique to CVID, the underlying hypogammaglobulinemia and attenuated EBV-specific T-cell responses in CVID may accentuate the amplitude and persistence of these fluctuations, sustaining chronic antigenic drive and a pro-inflammatory microenvironment that promotes lymphomagenesis.

### 4.3. Treatment Modifications

Our experience underscores the need to adapt treatment strategies for CVID-associated HL. The apparent benefit of bleomycin omission and the incorporation of targeted agents, such as BV, in our first patient should be interpreted with caution. Although the modified BV + AVD regimen appeared safe and resulted in durable remission, causality between treatment modification and clinical outcome cannot be inferred. The decision to omit bleomycin was guided by an individualized risk–benefit assessment, considering the patient’s underlying immunodeficiency and increased susceptibility to pulmonary toxicity. Current evidence supporting this approach in CVID-associated HL is limited to single-case experiences [25,26]. Rael et al. similarly reported successful use of BV with rituximab in an EBV-positive adult, underlining the effectiveness of antibody-based strategies [20].

It should be emphasized that the BV–AVD regimen itself carries a substantial risk of treatment-related infections. In the ECHELON-1 phase III trial, BV–AVD was associated with a higher incidence of febrile neutropenia and infection compared to ABVD, and primary prophylaxis with granulocyte colony-stimulating factor (G-CSF) is now recommended for all patients receiving this regimen. In our first case, G-CSF support was administered throughout therapy. Although bleomycin was omitted to mitigate pulmonary toxicity, there is no evidence that BV–AVD is better tolerated than ABVD or nivolumab-AVD in patients with immunodeficiency such as CVID. Consequently, the use of BV-based regimens in this setting should be regarded as an individualized, risk-adapted approach rather than an evidence-based standard [27,28].

While novel regimens such as brentuximab vedotin plus AVD (BV–AVD) and nivolumab plus AVD (Nivo–AVD) have demonstrated superior progression-free survival compared with ABVD in the general cHL population, these results derive primarily from the pivotal phase III HD21 (BreCADD) and SWOG S1826 (Nivo–AVD) trials. Both studies excluded patients with primary immunodeficiencies, limiting the generalizability of their findings to CVID-associated HL. Consequently, treatment in this population should remain highly individualized and multidisciplinary, integrating evidence from these landmark trials while accounting for the distinct infectious risks, immune dysregulation, and treatment-tolerability concerns inherent to CVID [29,30].

By contrast, our second patient required multiple salvage therapies, including BV, rituximab, bendamustine, and PD-1 blockade, but none achieved durable control. This parallels Ellwood’s case, where comorbidities and immune dysfunction undermined therapy [19]. Kishore et al. emphasized infection management without specifying lymphoma therapy, reflecting the challenges of balancing oncologic and infectious risks [18]. Pediatric cases used conventional regimens—reduced-dose ABVD or ABVD plus cyclophosphamide, vincristine, procarbazine, and prednisolone (COPP) —with favorable responses but without access to novel agents [21,22]. Aghamohammadi et al. reported two pediatric siblings who received 12 courses of MOPP (mechlorethamine, vincristine, procarbazine, prednisone) alternating with ABVD, but died from infectious complications despite initial remission, underscoring the poor tolerance of conventional chemotherapy in immunodeficient children [24]. Tatci’s patient received chemotherapy combined with radiotherapy, but succumbed to infectious complications, highlighting the risks of radiation in immunodeficient children [23]. Unlike Tatci’s report, we deliberately avoided radiotherapy in both patients to reduce infection-related morbidity, aligning with modern recommendations.

### 4.4. Targeted and Novel Therapies

Our cases also demonstrate the evolving role of targeted therapies. BV substitution for bleomycin was safe and effective in our first case, consistent with Rael’s experience. Rituximab, used by both Rael and in our second patient, provided only transient benefit and carries a risk of further immunosuppression [20]. Notably, our second patient represents one of the first reported uses of PD-1 checkpoint inhibition in CVID–HL. Although PD-1 blockade has shown efficacy in refractory HL [31], its safety in CVID is uncertain, as immune activation may exacerbate underlying dysregulation. The absence of checkpoint inhibitor use in all prior CVID–HL reports underscores both the novelty and the need for caution in this therapeutic approach.

Targeted therapies therefore represent promising, though double-edged, options in CVID-associated HL. BV is an effective alternative to bleomycin, rituximab may enhance efficacy but increases infectious risk, and PD-1 inhibitors, while highly active in HL, require careful selection and monitoring in immunodeficient patients. Their role in CVID remains to be clarified by future studies.

### 4.5. Outlook

Taken together, our cases, compared with the eight published reports, suggest three key conclusions. First, early diagnosis and timely IVIG initiation facilitate infection control and improve therapy tolerance, as demonstrated by our first patient and several pediatric cases, in contrast to late-diagnosed adults with poor outcomes. Second, EBV may play a more consistent role in CVID-associated HL than previously recognized, with both of our patients showing EBV positivity, in contrast to the variable or unspecified status in the literature. Third, treatment strategies require modification, including omission of bleomycin, avoidance of radiotherapy, and selective use of antibody-based or checkpoint therapies, balancing efficacy with infection risk. Management of HL in CVID must therefore be individualized and multidisciplinary, involving hematology, immunology, and infectious disease expertise.

These conclusions are concordant with the findings of a recent comprehensive review of immune-deficiency- and immune-dysregulation-associated EBV-positive HL, which identified this form as a distinct clinicopathologic entity characterized by frequent EBV positivity, impaired T-cell surveillance, and limited tolerance to standard chemotherapy [16]. The authors further highlighted that BV and PD-1 inhibitors may provide clinical benefit in this context but require vigilant infection control and multidisciplinary oversight—approaches that were also crucial in our management. Moreover, early recognition of underlying immunodeficiency and timely immunoglobulin replacement were emphasized as key determinants of outcome, aligning with the favorable course of our first patient.

While IVIG remains indispensable for infection prophylaxis [32,33,34], its role in lymphoma prevention remains unclear. Larger, multicenter studies are needed to establish evidence-based guidelines, clarify the role of EBV monitoring, and evaluate the safety of targeted therapies, particularly PD-1 inhibitors, in this vulnerable population.

Among these, the two pediatric sibling cases reported by Aghamohammadi et al. are noteworthy, as both developed CVID-associated HL within the same family, while their father and uncle also had HL [24]. This familial clustering suggests a possible genetic predisposition linking immune dysregulation and lymphomagenesis. Recent reports of monogenic CVID-like disorders involving CTLA4, LRBA, PIK3CD, and NFKB1 and 2 support this concept and highlight the importance of considering genetic testing in CVID–HL patients, especially when familial cases occur [35,36].

The comparative table (Table 1) below summarizes the clinical characteristics and treatment outcomes of HL cases in CVID patients, including the two presented cases and those reported in the literature. It illustrates the variability in EBV status, treatment responses, and outcomes, highlighting the need for individualized treatment approaches.

Currently, there are no dedicated clinical guidelines for the management of HL in patients with CVID. Treatment decisions therefore rely on standard HL protocols with necessary modifications to mitigate infection risk and treatment-related toxicity. Optimal care requires a multidisciplinary approach, incorporating hematology, immunology, and infectious disease expertise, with attention to IVIG replacement, infection surveillance, and EBV monitoring to improve outcomes.

This study has several limitations. Only two CVID–HL cases were identified (0.89% of 224 HL patients); this is consistent with the reported incidence of 0.46–1.4%, but restricts generalizability. The retrospective design introduces potential bias related to incomplete clinical data and evolving treatment practices. Furthermore, differences in disease course and therapeutic response between the two patients highlight the heterogeneity of CVID-associated HL. Larger, multicenter studies are needed to establish evidence-based guidelines and to clarify the role of immunoglobulin replacement, EBV monitoring, and targeted therapies in this rare population.

## 5. Conclusions

HL occurring in the context of CVID represents a rare but clinically significant entity characterized by marked heterogeneity, infectious complications, and suboptimal treatment tolerance. The two EBV-positive cases presented here expand the limited body of literature and illustrate that early recognition of CVID, proactive infection control, and carefully individualized therapy may improve clinical outcomes, although definitive causal relationships cannot be inferred. The potential role of targeted agents such as BV and immune checkpoint inhibitors appears promising but remains to be clarified in immunodeficient hosts.

Given the absence of disease-specific management guidelines, treatment of CVID-associated HL should rely on multidisciplinary collaboration between hematology, immunology, and infectious disease specialists, with therapy tailored to the patient’s immune status and comorbidities. Larger, multicenter studies and prospective registries are required to elucidate the contribution of EBV-driven mechanisms, optimize treatment algorithms, and determine the safety and long-term efficacy of novel targeted and immune-based approaches in this highly vulnerable population.

## Figures and Tables

**Table 1:** Comparison of our two cases with international reports of Hodgkin lymphoma (HL) in common variable immunodeficiency (CVID) patients, according to age, treatment strategy, and EBV status.

Case	Age	Treatment	EBV Status
Case 1 (Own)	33	BV + AVD → CMR	Positive
Case 2 (Own)	56	EBVD → DHAP → BV →PD-1-i + Rituximab → DHAC → BV + Bendamustine → Death	Positive
Rael et al. [20]	25	Rituximab + BV → CMR	Positive
Kishore et al. [18]	50	Not specified	Not specified
Ellwood et al. [19]	Not specified	Not specified	Not specified
Özdemir et al. [21]	9	Reduced-dose ABVD → CMR	Negative
Tootoonchi et al. [22]	13	ABVD + COPP → CMR	Negative
Tatci et al. [23]	7	AVD + RT → Death	Not mentioned
Aghamohammadi et al., Case 1 [24]	16	MOPP + ABVD → Death	Not mentioned
Aghamohammadi et al., Case 2 [24]	11	MOPP + ABVD → Death	Not mentioned

EBV: Epstein–Barr virus; BV: brentuximab vedotin; AVD: doxorubicin, vinblastine, and dacarbazine; EBVD: epirubicin, bleomycin, vinblastine, and dacarbazine; DHAP—dexamethasone, high-dose cytarabine and cisplatin; PD-1-i—programmed cell death protein 1 inhibitor; DHAC: dexame-thasone, cytarabine, carboplatin; ABVD: doxorubicin, bleomycin, vinblastine, and dacarbazine; CMR: complete metabolic remission; RT: radiation therapy; COPP: cyclophosphamide, vincristine, procarbazine, and prednisolone; MOPP: (mechlorethamine, vincristine, procarbazine, prednisone).

## Data Availability

Datasets generated during the current study are available from the corresponding author on reasonable request.

## Abbreviations

The following abbreviations are used in this manuscript:

Abbreviation	Full Term
ABVD	doxorubicin, bleomycin, vinblastine, and dacarbazine
AVD	doxorubicin, vinblastine, and dacarbazine
BV	brentuximab vedotin
CMR	complete metabolic remission
CMV	Cytomegalovirus
COPP	cyclophosphamide, vincristine, procarbazine, and prednisolone
CT	computed tomography
CTLA4	cytotoxic T-lymphocyte associated protein 4
CVID	Common Variable Immunodeficiency
DHAC	dexamethasone, cytarabine, carboplatin
DHAP	dexamethasone, high-dose cytarabine and cisplatin
EBV	Epstein–Barr virus
EBVD	epirubicin, bleomycin, vinblastine, and dacarbazine
HL	Hodgkin lymphoma
HLH	Hemophagocytic Lymphohistiocytosis
ICON	International Consensus Document
IgA, IgG, IgM	Immunoglobulin A, G, M
IVIG	intravenous immunoglobulin
LMP1/LMP2	latent membrane protein 1/2
LRBA	LPS-responsive beige-like anchor protein
MOPP	mechlorethamine, vincristine, procarbazine, prednisone
NFKB1/2	nuclear factor kappa-light-chain-enhancer of activated B cells 1 and 2
NHL	Non-Hodgkin Lymphoma
PD-1	programmed cell death protein 1
PD-1-i	programmed cell death protein 1 inhibitor
PD-L1/D-L2	programmed cell death-ligand 1/2
PET/CT	positron emission tomography–computed tomography
PIK3CD	phosphatidylinositol-4,5-bisphosphate 3-kinase catalytic subunit delta
PMR	partial metabolic response
RS Cells	Reed–Sternberg Cells
RT	radiation therapy
